# Urban-rural differences in a population-based breast cancer screening program in Croatia

**DOI:** 10.3325/cmj.2011.52.76

**Published:** 2011-02

**Authors:** Valerija Stamenić, Marija Strnad

**Affiliations:** 1 Department for Projects and Programmes, Directorate for Medical Affairs, Ministry of Health and Social Welfare, Zagreb, Croatia; 2 Andrija Štampar School of Public Health, Medical School, University of Zagreb, Zagreb, Croatia

## Abstract

**Aim:**

To investigate urban-rural differences in the distribution of risk factors for breast cancer.

**Methods:**

We analyzed the data from the first round of the “Mamma” population based-screening program conducted in Croatia between 2007 and 2009 and self-reported questionnaire results for 924 patients with histologically verified breast cancer. Reproductive and anthropometric characteristics, family history of breast cancer, history of breast disease, and prior breast screening history were compared between participants from the city of Zagreb (n = 270) and participants from 13 counties with more than 50% of rural inhabitants (n = 654).

**Results:**

The screen-detected breast cancer rate was 4.5 per 1000 mammographies in rural counties and 4.6 in the city of Zagreb, while the participation rate was 61% in rural counties and 59% in Zagreb. Women from Zagreb had significantly more characteristics associated with an increased risk of breast cancer (*P* < 0.001 in all cases): no pregnancies (15% vs 7%), late age of first pregnancy (≥30 years) (10% vs 4%), and the most recent mammogram conducted 2-3 years ago (32% vs 14%). Women from rural counties were more often obese (41% vs 28%) and had early age of first live birth (<20 years) (20% vs 7%, *P* < 0.001 for both).

**Conclusion:**

Identification of rural-urban differences in mammography use and their causes at the population level can be useful in designing and implementing interventions targeted at the reduction of inequalities and modifiable risk factors.

Significant differences in breast cancer frequency have been identified in different socioeconomic groups, ethnic groups, and between urban and rural populations (1,2). Living in rural areas may be associated with lower access to health care and mammography screening (3), as well as with late-stage diagnosis (4). This often means that patients need to travel great distances to receive care (5). Blair et al found that people in rural and urban areas were diagnosed with breast cancer at similar stages of the disease, although those from rural communities lacked basic cancer information because they did not have access to cancer education programs offered in urban areas (6). Robbins et al explained the higher breast cancer incidence in the San Francisco Bay Area than in other regions by known risk factors: parity, age at first full-term pregnancy, breast-feeding, age at menarche, and age at menopause (7). In Croatia, Polašek et al found that in a period without a national cancer screening program access to health care was the strongest cancer screening utilization predictor in adult rural population (8).

Risk factors for breast cancer are mostly those related to the reproductive life of women (9,10): menarche, nulliparity or late age at first birth, late menopause, as well as hormonal factors, be they endogenous or exogenous (eg, term use of oral contraceptives or menopausal hormonal replacement). Other risk factors related to hormonal status include obesity and a diet characterized by a high caloric intake, low intake of fruits and vegetables, and lack of physical activity (11). Radiation, in particular during breast development, was also found to be a risk factor (12), while the role of contaminants, such as xenoestrogens and certain pesticides, remains controversial. Four- to 5-fold risk of developing breast cancer was associated with epithelial proliferative lesions, particularly atypical ductal or lobular hyperplasia (11).

In Croatia, breast cancer is the leading cancer among women, amounting to 27% of new female cancer cases; moreover, the incidence rate in 2007 was 17% higher than in the previous year (13). In 2007, cancer incidence by county and age-standardized rates per 100 000 women varied considerably: from 273.1 (Šibensko-kninska county) to 437.7 (the city of Zagreb), but the prevalence of breast cancer risk factors remains unknown. A government-funded mammography screening program was established in October 2006 and has since been implemented in 21 counties, including the city of Zagreb (14).

Population-based screening for breast cancer is conducted through mammographic examination of all women of a specified age at prescribed time intervals. The implementation of population-based screening requires technical resources and trained personnel for double reading of mammograms, as well as a major media campaign (15).

Within a more extensive study of breast cancer risk factors, this study investigated urban-rural differences in reproductive, anthropometric, and family history of breast cancer and personal history of breast disease among women aged 50-69 from 13 rural counties and the city of Zagreb who participated in the first round of population-based mammography screening in Croatia.

## Materials and methods

### “Mamma” screening program

Organized population-based screening program in Croatia started in November 2006 on a target population of women aged 50-69. Coordinators from the Public Health Institutes from 21 counties distributed the invitations and coordinated the program at the county level. A separate database was formed for each county and the central unit had access to each of these databases through a common server located at the Croatian Ministry of Health and Social Welfare. The program is centrally coordinated by the Croatian National Institute of Public Health and includes 81 mammography units and more than 200 radiologists. Double reading is obligatory; if the result is normal, women are sent a letter of invitation to another routine screening in 2 years. If the result is abnormal, women and their family physicians are informed about the need for further assessment (14).

We used the Organisation for Economic Co-operation and Development definition of rural and urban from the Croatian Rural Development Strategy 2008-2013 of the Croatian Ministry of Agriculture, Fisheries, and Rural Development (16). Rural counties were considered those with a population density of 150 people or fewer per square mile and more than 50% of rural inhabitants (16). Out of 21 counties, we identified 13 rural counties with a total population of 1 926 219 people: Bjelovarsko-bilogorska, Brodsko-posavska, Karlovačka, Koprivničko-križevačka, Krapinsko-zagorska, Ličko-senjska, Požeško-slavonska, Sisačko-moslavačka, Šibensko-kninska, Virovitičko-podravska, Vukovarsko-Srijemska, Zadarska, and Zagrebačka county. From the group of urban counties, we selected the county of city of Zagreb (population: 779 145), which is the county with the highest population density. We excluded 5 counties with fewer than 50% rural inhabitants and Varaždin county due to lack of data (16).

### Participants

The first round of the “Mamma” screening program included 80 092 women aged 50-69 from the city of Zagreb and 184 425 from 13 rural counties. Participation in the screening program was free and based on an invitation. Of 204 352 women screened from 2007-2009, 924 were found to have breast cancer. Of these, 270 were from the city of Zagreb and 654 from rural counties: Bjelovarsko-bilogorska, 56; Brodsko-posavska, 64; Karlovačka, 42; Koprivničko-križevačka, 47; Krapinsko-zagorska, 41; Ličko-senjska, 16; Požeško-slavonska, 32; Sisačko-moslavačka, 45; Šibensko-kninska, 31; Virovitičko-podravska, 30; Vukovarsko-srijemska, 96; Zadarska, 70; and Zagrebačka, 84.

Screening was performed in women who lacked breast physical examination abnormalities, including nipple discharge, lumps, or thickening. Women with previous breast cancer and women who had not answered the questions addressing the studied risk factors were excluded.

*Questionnaire.* Women involved in the program were sent the questionnaire with an invitation letter to their home address. The invitation list was generated based on records of the Croatian Health Insurance Institute and the Ministry of Interior Affairs. The oldest women invited into the program were born between 1937 and 1941. The participants at the time of mammography completed the questionnaire and reported their age, age at menarche, number of pregnancies and deliveries, age at first live-birth, history and duration of breast feeding, use and duration of use of birth control pills (oral contraceptive), use and duration of hormone replacement therapy, menopausal status, age at menopause, personal or family history of breast cancer (defined as having first-degree or second-degree relative with breast cancer), breast symptoms (pain, tenderness, and swelling), breast procedures, weight, height, and the time of their last mammogram. To increase the response rate, reminders were made by telephone and field nurses motivated women to attend the screening.

BMI was calculated as self-reported current weight in kilograms divided by height in meters squared (kg/m^2^) and divided into three categories: lean weight (BMI≤25), overweight (25<BMI<30), and obesity (BMI≥30) (17).

The patients gave informed consent when they filled in the questionnaire. The study was approved by the Ethics Committee of the Medical School, University of Zagreb.

### Statistical analysis

Descriptive analysis and tabulations for all variables were performed using χ^2^ test with Yates correction when necessary and two-sided *t*-tests to compare predictive variables for urban-rural differences (18,19). Statistical calculations were performed using Statistica, version 9.0 (StatSoft, Tulsa, OK, USA).

## Results

**Figure 1:**
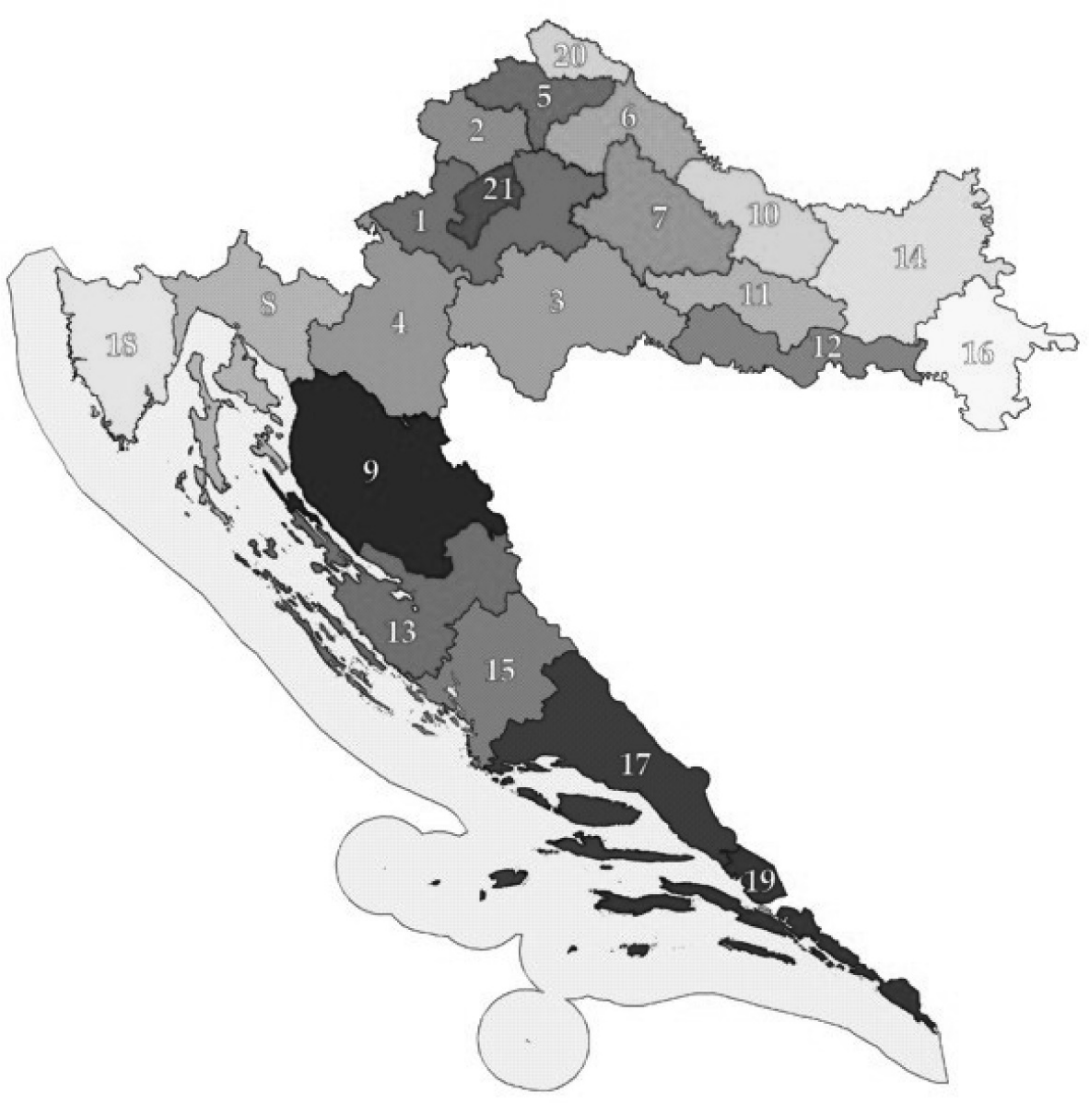
The map of Croatia with 21 counties. 1 – Zagrebačka; 2 – Krapinsko-zagorska; 3 – Sisačko-moslavačka; 4 – Karlovačka; 5 – Varaždinska (excluded due to lack of data); 6 – Koprivničko-križevačka;7 – Bjelovarsko-bilogorska; 8 – Primorsko-goranska*; 9 – Ličko-senjska; 10 – Virovitičko-podravska; 11 – Požeško-slavonska; 12 – Brodsko-posavska; 13 – Zadarska; 14 – Osiječko-baranjska*; 15 – Šibensko-kninska; 16 – Vukovarsko-srijemska; 17 – Splitsko-dalmatinska*; 18 – Istarska*; 19 – Dubrovačko-neretvanska*; 20 – Međimurska*; 21 – City of Zagreb. Asterisk indicates excluded counties with <50% rural inhabitants.

The first round of the population-based screening program included 204 352 women: 146 110 from 13 rural counties and 58 242 from the city of Zagreb (Figure 1, Table 1). We identified 924 women with breast cancer: 654 (0.45%) from rural areas and 270 (0.46%) from Zagreb. The detected cancer rate was 4.5 per 1000 mammographies in rural counties and 4.6 per 1000 mammographies in Zagreb (Table 1).

**Table 1:** The rate of screen-detected breast cancer per 1000 mammographies in 13 rural counties and the city of Zagreb in the first round of the “Mamma” screening program (2007-2009)

County of residence	Participating women, N (%)	Screened women	Screen-detected cancers	Cancer/per 1000 mammographies
Rural counties:	18 4 425 (60.6)	146 110	654	4.5
Vukovarsko-srijemska	18 446 (59.1)	15 122	96	6.3
Brodsko-posavska	14 112 (53.3)	12 406	64	5.1
Bjelovarsko-bilogorska	16 164 (78.1)	12 061	56	4.6
Zadarska	20 906 (69.9)	15 299	70	4.6
Zagrebačka	21 355 (47.0)	18 203	84	4.6
Koprivničko-križevačka	11 642 (63.8)	10 633	47	4.4
Požeško-slavonska	10 312 (74.6)	7296	32	4.4
Ličko-senjska	4880 (47.9)	3451	16	4.4
Virovitičko-podravska	9044 (61.1)	6938	30	4.3
Karlovačka	12 436 (51.2)	9662	42	4.3
Krapinsko-zagorska	13 438 (68.3)	11 598	41	3.5
Sisačko-moslavačka	18 998 (54.9)	13 712	45	3.3
Šibensko-kninska	12 692 (59.2)	9729	31	3.2
Urban county				
City of Zagreb	80 092 (58.5)	58 242	270	4.6
Total	264 517 (59.6)	204 352	924	4.5

The screening program involved 66dedicated screening facilities (47 in rural counties and 19 in Zagreb), with specialized equipment and trained staff who performed screening and/or for further assessment in cases when an abnormality was detected. There were only slight rural-urban differences in the number of screening facilities per 10 000 invited women when all rural counties were considered, although there were great differences between individual rural counties, from 0.8 in Brodsko-posavska county to 3.9 in Ličko-senjska county (Table 2).

**Table 2:** The number and rate of dedicated screening facilities per 10 000 invited women in 13 rural counties and the city of Zagreb in the “Mamma” screening program

County of residence	Invited women	Screening facilities	Rate of screening facility per 10 000 invited women
Rural counties:	310 415	47	1.6
Ličko-senjska	10 176	4	3.9
Zagrebačka	45 421	11	2.4
Virovitičko-podravska	14 779	3	2.0
Krapinsko-zagorska	19 657	4	2.0
Šibensko-kninska	21 256	4	1.9
Požeško-slavonska	13 815	2	1.4
Sisačko-moslavačka	34 649	4	1.2
Koprivničko-križevačka	18 253	2	1.1
Zadarska	29 889	3	1.0
Bjelovarsko-bilogorska	20 675	2	1.0
Vukovarsko-srijemska	31 113	3	1.0
Karlovačka	24 289	3	1.0
Brodsko-posavska	26 443	2	0.8
Urban county			
City of Zagreb	136 261	19	1.4
Total	446 676	66	1.5

The questionnaires were collected at the time of mammography screening for 913 of the 924 women (99%) (Table 3). Compared with women from rural counties, significantly more women from Zagreb had the characteristics that increased the risk of breast cancer: no pregnancies (15% vs 7%), late age of first pregnancy (≥30-year) (10% vs 4%), and the last mammogram conducted 2-3 years ago (32% vs 14%) (*P* < 0.001 for all comparisons). Compared with women from Zagreb, women from rural counties were more often obese – BMI>30 (41% vs 28%, respectively) and had early age of first live birth (<20 years) (20% vs 7%, *P* < 0.001 for both comparisons, Table 3).

**Table 3:** Characteristics of women with screen-detected breast cancer who participated in the first round of population-based screening in Croatia, 2007-2009*

Characteristic	Breast cancer patients, n (%)		df	χ^2^	*P^†^*
	rural	urban			
Age at screening, years:	654 (100.0)	268 (99.3)	3	0.2487	0.969
50-54	91 (13.9)	40 (14.8)			
55-59	150 (22.9)	61 (22.6)			
60-64	143 (21.9)	60 (22.2)			
65-69	270 (41.3)	107 (39.6)			
mean age±SD	58.3 ± 11.4	61.2 ± 9.4			
Age at menarche, years:	635 (97.1)	267 (98.9)	2	6.226	0.044
<12	38 (5.8)	26 (9.6)			
12-13	251 (38.4)	115 (42.6)			
≥14	346 (52.9)	126 (46.7)			
mean age±SD	13.7 ± 3.6)	13.5 ± 3.5			
Current menstrual status:	647 (98.9)	267 (98.9)	1	0.5584	0.455
pre-menopausal	37 (5.6)	12 (4.4)			
peri/postmenopausal	610 (93.3)	255 (94.4)			
Age at menopause, years:	598 (91.4)	252 (93.3)	3	4.0369	0.257
<45	75 (12.3)	36 (14.1)			
45-49	159 (26.1)	57 (22.3)			
50-54	296 (48.5)	138 (54.1)			
≥55	68 (11.1)	21 (8.2)			
mean age±SD	49.0 ± 6.8	46.7 ± 6.9			
Use of OC (ever):	652 (99.7)	267 (98.9)	1	1.2244	0.268
no	538 (82.3)	212 (78.5)			
yes	114 (17.4)	55 (20.4)			
Duration of OC use, years:	114 (17.4)	53 (20.4)	2	4.5337	0.104
<5	64 (55.4)	35 (63.6)			
5-9	32 (28.6)	7 (12.7)			
≥10	18 (16.1)	11 (20.0)			
mean months±SD	52.6 ± 7.9	50.4 ± 7.4			
Use of HRT (ever):	652 (99.7)	267 (98.9)	1	9.5575	0.002
no	593 (90.9)	224 (83.0)			
yes	59 (9.0)	43 (16.0)			
Duration of HRT use, years:	59	43	2	1.0429	0.594
<5	19 (32.2)	18 (41.9)			
5-9	25 (42.4)	15 (34.9)			
≥10	15 (25.4)	10 (23.2)			
mean months±SD	78.8 ± 8.3	74.7 ± 8.4			
Pregnancy history:	644 (98.5)	267 (98.9)	1	14.2571	<0.001
never pregnant	45 (6.9)	40 (14.8)			
one pregnancy	113 (17.3)	43 (15.9)			
≥2 pregnancies	486 (74.3)	184 (68.1)			
median, range	2.0 (1-20)	2.0 (1-7)			
Parity:	640 (97.8)	267 (98.9)	2	21.6220	<0.001
nulliparous	48 (7.3)	41 (15.2)			
uniparous	134 (20.5)	74 (27.4)			
multiparous	458 (70.0)	152 (56.3)			
median (range)	2.0 (1-7)	2.0 (1-4)			
Age at first live birth, years:	592 (90.5)	255 (94.4)	3	45.4571	<0.001
nulliparous	48 (7.3)	41 (15.2)			
<20	132 (20.2)	20 (7.4)			
20-29	388 (59.3)	166 (61.5)			
≥30	24 (3.7)	28 (10.4)			
mean age±SD	20.4 (4.6)	24.8 (4.9)			
Period of breastfeeding, months:	632 (96.6)	259 (95.9)	2	11.5929	0.003
0 (include no live birth)	108 (16.5)	60 (22.2)			
≤12	387 (59.2)	166 (61.5)			
>12	137 (20.9)	33 (12.2)			
mean months±SD	10.8 ± 3.2	8.8 ± 3.0			
Family history of breast cancer:	652 (99.7)	255 (94.4)	2	8.1097	0.017
none	579 (88.5)	222 (82.2)			
second degree	30 (4.6)	16 (5.9)			
first degree	43 (6.6)	32 (11.8)			
History of benign disease:	651 (99.5)	253 (93.7)	1	7.1417	0.007
no	568 (85.5)	203 (75.2)			
yes	83 (12.7)	50 (18.5)			
Body mass index:	632 (96.6)	270 (100)	2	21.3414	<0.001
<25	100 (15.0)	69 (25.5)			
25.0-29.9	261 (40.2)	125 (46.3)			
≥30	271 (41.4)	76 (28.1)			
median weight (kg), range	78.0 (40-140)	75.5 (48-115)			
Height, cm	630 (96.3)	270 (100)	3	0.5630	0.905
<159	151 (23.3)	67 (28.1)			
160-164	192 (29.6)	76 (28.1)			
165-169	178 (27.1)	77 (28.5)			
>170	109 (16.2)	50 (18.4)			
mean±SD	163.4 (12.4)	163.9 (12.8)			

*Abbreviations: OC – oral contraceptive; SD – standard deviation; HRT – hormonal replacement therapy; df – degrees of freedom.

†χ^2^ used to test categorical variables for statistical significance; *t*-tests were used on continuous variables.

**Figure 2:**
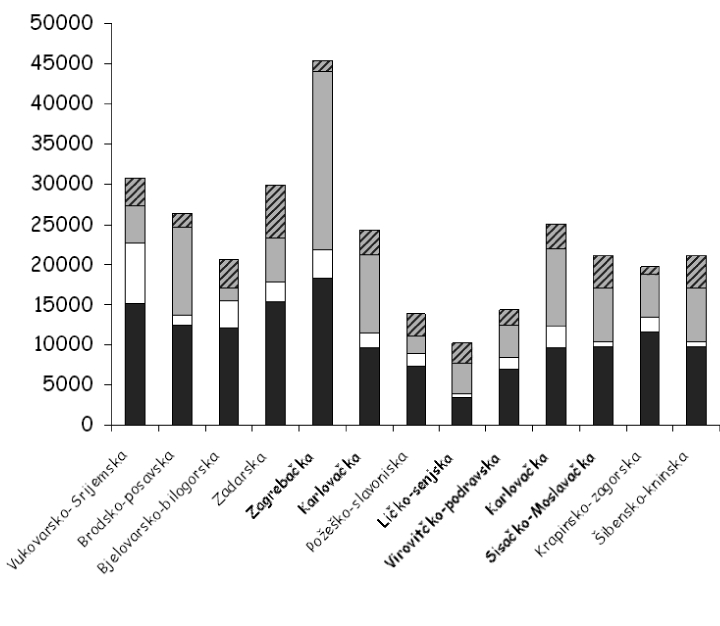
Differences between counties among invited women in the first round of breast cancer screening in Croatia, 2007-2009. Black – number of screened women; gray – women who did not attend screening; white – women who performed mammography in the past 12 months; diagonal lines – deceased women, women with wrong address, and women temporarily out of Croatia.

The participation rate was 59% in the city of Zagreb and 61% in rural counties, ranging between 47% in Zagrebačka county and 78% in Bjelovarsko-bilogorska county (Table 1). The number of screened women and reasons for non-compliance with breast cancer screening recommendations varied between urban and rural counties (Table 4) and from county to county (Figure 2).

**Table 4:** The number of screened women and reasons for non-compliance with screening program after receiving an invitation, 2007-2009

	No. (%) of women from	
	rural counties	city of Zagreb
Screened women	146 110 (47.1)	58 242 (42.7)
**Reasons for non-compliance:**		
mammography <12mo	16 261 (5.2)	15 673 (11.5)
already receiving therapy	5482 (1.8)	303 (0.2)
other reason	7851 (2.5)	1014 (0.7)
deceased	4336 (1.4)	877 (0.6)
temporarily out of place of residence	7673 (2.5)	550 (0.4)
not-attended	122 702 (39.5)	59 602 (43.7)
Invited women	310 415 (100.0)	136 261 (100.0)

Women with breast cancer in Zagreb had lower parity than women in rural counties (91% vs 85%; *P* < 0.001). They also had significantly greater median age of first live birth (25 vs 19 years; *P* < 0.001) (Table 3).

Most of the women with breast cancer in our study were postmenopausal (93% of rural and 94% of urban women; *P* = 0.455) (Table 3). There was no urban-rural difference in mean age at menarche (13.7 years in rural and 13.5 years in urban women; *P* = 0.044) and at menopause (49 years in rural and 47 in urban women; *P* = 0.257). Fifteen percent of urban women and 7% of rural women had never been pregnant (*P* < 0.001; Table 3).

More women in rural counties had an average BMI>30 (41% vs 28%; *P* < 0.001) and there was no difference in height between urban and rural women (163.4 vs 163.9 cm, respectively) (*P* = 0.905) (Table 4).

Breast feeding was reported by 80% of rural women and 74% of urban women (*P* = 0.039), and more rural women breast fed for longer than 12 months (21% vs 12%; *P* = 0.003) (Table 4).

There was no difference in the prevalence of postmenopausal breast cancer patients between rural and urban women (93% vs 94%, respectively; *P* = 0.455) (Table 3). The history of hormonal replacement therapy use was more common among urban than among rural women (16% vs 9%, respectively; *P* = 0.003) but there was no difference in the history of oral contraceptive use (20% in urban women vs 17% in rural women; *P* = 0.338). Significantly more rural women had early age of the first live birth (<20 years) (20% vs 7%; *P* < 0.001). There was a significant urban-rural difference in having performed a mammogram in the last 2 years (31% vs 14%, respectively; *P* < 0.001) (Table 5). More urban than rural women had a first-degree relative with breast cancer history (12% vs 7%; *P* = 0.011), and similar number of women from both groups had a second degree relative with breast cancer history (5% of rural vs 6% of urban women; *P* = 0.493) (Table 5).

**Table 5:** The proportions of breast cancer risk factors among 924 patients with detected breast cancer who participated in the first round of the “Mamma” program

	No. (%) of breast cancer patients				
Characteristics	rural (n = 654)	urban (n = 270)	Difference (95%confidence interval)	χ^2^	*P*
Body mass index >30 (kg/m^2^)	271 (41.4)	76 (28.1)	13.31 (6.52 to 19.66)	13.403	<0.001
Menarche:					
<12	38 (5.9)	26 (9.7)	3.45 (0.45 to 7.34)	3.058	0.080
≥14	346 (52.9)	126 (46.5)	6.30 (-0.77 to 13.37)	2.778	0.095
Parity	590 (90.3)	231 (85.6)	4.76 (-0.01 to 9.53)	3.928	0.047
First live birth <20 y	132 (20.2)	20 (7.4)	12.78 (8.4 to 17.16)	21.794	<0.001
Breast-feeding	524 (80.1)	199 (73.7)	6.42 (0.34 to 12.5)	4.258	0.039
Exogenous hormones:					
oral contraceptive use (% ever)	114 (17.4)	55 (20.4)	2.94 (-2.67 to 8.5)	0.917	0.338
hormonal replacement therapy use (% ever)	59 (9.0)	43 (15.9)	6.90 (-2.01 to 11.78)	8.577	0.003
Breast cancer history:					
in first-degree relative	43 (6.5)	32 (11.8)	5.28 (0.98 to 9.58)	6.456	0.011
in second-degree relative	30 (4.6)	15 (5.9)	1.34 (-1.9 to 4.58)	0.470	0.493
Prior screening before 2-3 y	94 (14.4)	85 (31.5)	17.11 (10.95 to 23.27)	34.735	<0.001
Breast symptoms	126 (19.2)	50 (18.6)	0.62 (4.92 to 6.16)	0.016	0.899

## Discussion

Our study investigated urban-rural differences in the distribution of risk factors for breast cancer among women participating in the first round of the population-based screening program in Croatia. We found that women from Zagreb had significantly more characteristics associated with Western lifestyle that increased the risk of breast cancer (20), including no pregnancies, late age of first pregnancy (≥30 years), and mammographic examination in the last 2-3 years, while women from rural counties were more often obese and had younger age of first live birth (<20 years).

Possible explanations for the less frequent use of preventive services in rural counties than in Zagreb include greater distances to medical facilities, less access to services, and lower socioeconomic status (1). Indeed, we found that older age, living temporarily out of the place of residence, and greater distance from health services may be significant barriers to the use of preventive health care services in rural areas. Economic concerns were not relevant to our study, since all women were invited to the free screening regardless of whether they were insured.

We found a similar level of screen-detected breast cancer rate per 1000 mammographies (4.6 vs 4.5) when all rural counties were considered, although our findings, as well as previous cancer incidence data in Croatia (13), showed great variation among rural counties, from 3.2 in Šibensko-kninska county to 6.3 in Vukovarsko-srijemska county.

After the first invitation round, the participation rate in Croatia stabilized at around 60%, which is lower than the 70% rate that the EU Guidelines for Quality Assurance in Mammography Screening recommend as acceptable and the 75% rate that they recommend as desirable (21). The coverage varies considerably from program to program and from country to country (21-23). Attendance is naturally a strong predictor of the program’s impact, and the attendance rate of 61% in rural counties and 59% in Zagreb indicates that the program is well accepted, with no urban-rural gradient in screening participation, but efforts have to be taken to achieve desirable participation rate (14). The participation rate showed a considerable variation between the counties, from 48% in Ličko-senjska county to 78% in Bjelovarsko-bilogorska county. This could be explained by the fact that Ličko-senjska county has more older residents and residents living temporarily out of their permanent place of residence and far from mammography service facilities. Our results are similar to the study by McElroy, who found no significant difference in early detection of cancer between urban and rural communities (1). On the other hand, the Norwegian Breast Cancer Screening program showed that rural areas had a greater attendance rate than Oslo (90% vs 79%), which most probably reflected different access to private mammography services (23). Screening attendance might be affected by false-positive mammography and overdiagnosis in organized mammography screening (24,25).

There are no uniformly accepted definitions of rural and urban areas and this makes the comparison between studies difficult (26). Also, within a single county there are heterogeneous populations and environments, which is likely to mask trends at smaller geographical levels. However, a few studies have evaluated patterns of urban/rural risk even at such levels (27,28). Future studies should examine rural/urban differences in conjunction with other risk factors at different geographical levels, such as neighborhood block, tract, or city (29).

Contrary to our findings, Chelpin et al (23) found a higher coverage in the mixed urban-rural area in Fyn (20%) than in Copenhagen; Thurfjell et al (30) found a lower participation rate in Stockholm than in rural Sweden; and Vizcaino et al (31) found a lower participation rate in Valencia than in Navarra.

On the other hand, Blair et al found substantial differences in the distribution of breast cancer and probable risk factors (parity, age at first full-term pregnancy, breast-feeding, age at menarche, age at menopause, and alcohol consumption) between the urban San Francisco Bay Area and rural regions (6). Increased breast cancer incidence rate in the San Francisco Bay Area could be completely accounted for by regional differences in known risk factors (2). Studies on migration, acculturation, and breast cancer incidence demonstrate that incidence rates increase in women who migrate from low-incidence to high-incidence countries (32).

Our study found a significantly higher proportion of obesity among postmenopausal rural women (BMI≥30), which is in accordance with previous results (33-39). This difference may be explained by a high-fat diet and lower socio-economic status in rural women (40).

A family history of breast cancer has long been recognized as a risk factor for the disease, and the risk of developing breast cancer is increased 1.5- to 3-fold if a woman has mother or sister with breast cancer (41). Our study found a higher proportion of first degree relatives with breast cancer among urban women. These women are at higher risk of breast cancer than general population because of shared genetic factors and possibly because of shared exposures to environmental and lifestyle factors (42). The recent identification of common genetic variants, however, has not heralded the arrival of personalized prevention measures of breast cancer, although it has been recommended that these women undergo annual mammography screening beginning with the age of 40 years (43).

Our findings suggest that mammographic screening has played a major role in the increase in incidence of breast cancer in Croatia (13), but the increase had started well before the screening became widely available (44). The increasing trends observed before 1995 can be attributed to greater disease awareness, greater detection by physical breast examination (either self-examination or examination by physician or a nurse), changes in reproductive factors, increasing use of hormone treatment after menopause, and increasing rates of obesity (39).

Epidemiological studies have consistently identified a number of breast cancer risk factors associated with increased exposure to endogenous estrogens (41,43-47). Our findings suggest that the observed differences between urban and rural women could be substantially reduced by changing the lifestyle, reducing obesity, and promoting breast feeding. It is important to educate the public and health care professionals in order to promote mammography screening (48,49), including the “Mamma” program. Finally, obesity, which increases the risk of many adverse health conditions including breast cancer, needs to be addressed through effective community interventions.

This study has several strengths: population-based design, the response rate of 99%, and availability of information on many established and probable risk factors that may influence breast cancer, with no recall bias. However, one of its limitations is that the questionnaire did not distinguish among types of hormone replacement therapy. Also the data on socioeconomic status, such as education, income, type of occupation, and in some populations, ethnicity, and religion were missing (32). Besides this, the data were self-reported and therefore not verified by objective observers. Future studies should take into account both the women's attitude toward screening and the consistency of women's behavioral pattern (23,50-52). In addition, most research examines rural-urban residence at the time of the diagnosis, but does not examine exposures at critical life stages. Future research should examine residential history to analyze the critical exposures or timing of exposures that lead to greater breast cancer incidence.

In conclusion, our study identified several reproductive and anthropometric risk factors for breast cancer that are modifiable and can reduce inequalities between urban and rural areas. Although there are some effective programs that may reduce some of the preventable risk factors, the availability of these programs may need to be improved in several remote rural areas. Future studies should implement the Gail breast cancer risk prediction model (53,54) to explore how the distribution of established risk factors could explain the high incidence of breast cancer in some counties (Dubrovačko-neretvanska, Zadarska, Primorsko-goranska, and Istarska county) but not in other (Krapinsko-zagorska, Šibensko-kninska, Virovitičko-podravska, and Zagrebačka county). The current county differences in breast cancer incidence may reflect differences in risk factor prevalence but also differences in screening mammography use.
